# Asthma phenotypes in primary care

**DOI:** 10.1038/s41533-020-0170-6

**Published:** 2020-04-06

**Authors:** Persijn J. Honkoop, Niels H. Chavannes

**Affiliations:** 1 Department of Public Health and Primary Care, Leiden University Medical Centre, Leiden, The Netherlands; 2 National eHealth Living Lab (NeLL), Leiden, The Netherlands

**Keywords:** Asthma, Outcomes research

Asthma is a very common disease with a diverse manifestation of symptoms. Some people with asthma hardly have any symptoms, others have a high symptom load, and there are those who seem all fine and suddenly have a (very) severe exacerbation. Besides heterogeneity in symptoms, response to treatment varies widely too. All these varying factors make asthma a complicated disease to manage. Even though this has been known for a long time, up until quite recently most international guidelines advocated a rather one-size-fits-all approach to the management of asthma. However, this all changed with the advent of treatable traits. Now, recent international guidelines promote the identification and subsequent specific management of these treatable traits in people with asthma^1–5^. Treatable traits can be further subdivided into pulmonary, extrapulmonary, and lifestyle-related traits^3,6^.

The entire concept of a subdivision in different types of asthma is, of course, nothing new. In fact, we have been debating about distinctions ever since Orie introduced the Dutch Hypothesis in 1961 (which used to include even chronic obstructive pulmonary disease as a sort of subtype of asthma)^6^. What is different now though is that with the biologicals, we actually have new, specified treatments as well. Also, we have seen the arrival of all sorts of new tests (including genomics and metabolomics) and biomarkers, which allow for further precision management^7–10^.

All of this is good news, especially for people with severe asthma. However, depending on which guideline or article you pick, you can end up with somewhere between 10 and 25 different treatable traits and each have their own specific test(s)^4–6,8–10^. To try and find all these traits, by doing all these tests, places a large burden on a patient. Unfortunately, it is also simply unaffordable for society as a whole, considering the costs of all these tests. Most importantly, it is also unnecessary, since most people with asthma have a low symptom burden and few exacerbations, especially in primary care^11^. Therefore, it would be very useful if we could come up with an easy way to make a distinction in our primary care asthma population, between people with more or less severe asthma. Which is exactly what Kisiel et al. provide us with in their Article^12^.

In their well-performed study, they have assessed the data from 1291 individuals by a cluster analysis. This resulted in three clearly distinct asthma phenotypes with their own disease severity profile. Then, they validated their results in an independent cohort of another 748 people with asthma. The most severe phenotype, with the worst symptom score, worst quality of life, and highest exacerbation rate, was labeled early onset predominantly female phenotype. The best scores in terms of disease severity were for the adult onset predominantly male phenotype. In clinical practice, fitting a patient sitting in front of you into any of these three distinctive phenotypes gives an idea of what to expect in terms of disease severity

This is not the first study into phenotypes in primary care. The best known is the one by Haldar et al.,^13^ which showed four different phenotypes mainly based on distinctions in symptom load and inflammation. Unfortunately, that study did require sputum counts and peak-flow variability, both quite burdensome for the patient. Khusial et al.^14^ presented an article in our journal, which showed five different phenotypes of asthma. They also showed long-term outcomes for each of these. However, some of their phenotypes were small in numbers, which reduces external validity. Ortega et al.^15^ also performed a cluster analysis in primary care data, but they were more focused on describing different asthma exacerbation phenotypes. Finally, Metting and colleagues^16^ produced a diagnostic algorithm for obstructive airway diseases, based on data from 9297 patients, which could be used prior to the one described in this article, to distinguish between different obstructive diseases. The key advantages of the trial presented here by Kisiel et al.^12^ compared to these other studies are its numbers combined with the use of real-world data. By establishing the different phenotypes in data from over 1200 people and then validating them in another 748, they are firmly rooted and unlikely to be very different when applied to your patients. What is more (nearly), all of the parameters required to determine someone’s phenotype can be automatically available in the Electronic Health Record (EHR). In some countries this might not be a possibility yet, but with the advancement of EHRs it will definitely come in the future. This allows for potential automated warning signals for the more severe phenotype.

There are also some limitations to the study. The first one is the lack of disease outcomes as part of the cluster analysis. Instead of adding, for example, the level of asthma control as one of the parameters for forming different clusters, the authors opted to leave out disease outcomes and only assessed these once the clusters were formed. This is defendable, because it provides important information about the clusters. However, it does go against common clinical reasoning, where level of control, medication usage, and the amount of exacerbations someone had in the previous year(s) would be important information to determine someone’s disease severity. Another limitation is the lack of long-term outcomes. In other words, it is unknown how stable the phenotypes are over time with regard to outcomes and it might be that the adult onset predominantly male phenotype, for instance, might become more severe a couple of years down the line. The authors therefore suggest to regularly repeat the analysis, which can be burdensome for a patient. Fortunately, the assessment consists largely of data that hardly changes over time (allergy status, sex, age of onset, several comorbidities). Also, as mentioned earlier, most of this could be automated within an Health Electronic Record.

Overall, the results from the study by Kisiel et al.^12^ provide us with a relatively easy-to-use way of distinguishing our primary care asthma patients. This will allow us to identify those with a more severe profile, and subsequently we can then decide to follow these up more regularly, as the authors suggest. An added bonus is that we might also use it to decide which patients are suitable candidates to look into a bit further with regards to treatable traits, thus enhancing our ability to perform precision medicine.
